# Small Molecule Epigenetic Modulators in Pure Chemical Cell Fate Conversion

**DOI:** 10.1155/2020/8890917

**Published:** 2020-10-20

**Authors:** Zhao-Di Yuan, Wei-Ning Zhu, Ke-Zhi Liu, Zhan-Peng Huang, Yan-Chuang Han

**Affiliations:** ^1^Department of Cardiology, Center for Translational Medicine, Institute of Precision Medicine, The First Affiliated Hospital, Sun Yat-sen University, Guangzhou, China; ^2^Grade 19, Sun Yat-sen University Zhongshan School of Medicine, Sun Yat-sen University, Guangzhou, China; ^3^NHC Key Laboratory of Assisted Circulation (Sun Yat-sen University), Guangzhou, China

## Abstract

Although innovative technologies for somatic cell reprogramming and transdifferentiation provide new strategies for the research of translational medicine, including disease modeling, drug screening, artificial organ development, and cell therapy, recipient safety remains a concern due to the use of exogenous transcription factors during induction. To resolve this problem, new induction approaches containing clinically applicable small molecules have been explored. Small molecule epigenetic modulators such as DNA methylation writer inhibitors, histone methylation writer inhibitors, histone acylation reader inhibitors, and histone acetylation eraser inhibitors could overcome epigenetic barriers during cell fate conversion. In the past few years, significant progress has been made in reprogramming and transdifferentiation of somatic cells with small molecule approaches. In the present review, we systematically discuss recent achievements of pure chemical reprogramming and transdifferentiation.

## 1. Introduction

In 1958, Gurdon et al. first reported unknown factors in the oocyte cytoplasm could reprogram differentiated cells to a pluripotent state [1]. The breakthrough suggested that somatic cells are flexible and could be converted to other cell types. In 1987, Davis et al. discovered that a single transcription factor, MyoD, was able to induce fibroblasts directly into myoblasts, which indicated only a few transcription factors could make cell fate decisions [2]. Nearly 20 years later, Yamanaka's team found that pluripotent stem cells (iPSCs) could be obtained from somatic cells using four key transcription factors (Oct4, Sox2, Klf4, and c-Myc, termed OSKM) [3]. One year later, two research groups independently succeeded in creating human iPSCs using a similar method [4, 5]. With this new iPSC technology, the molecular mechanisms of cell fate transition could be investigated and diverse applications, including drug screening, disease modeling, and cell therapy, could be developed [6].

**Figure 1:**
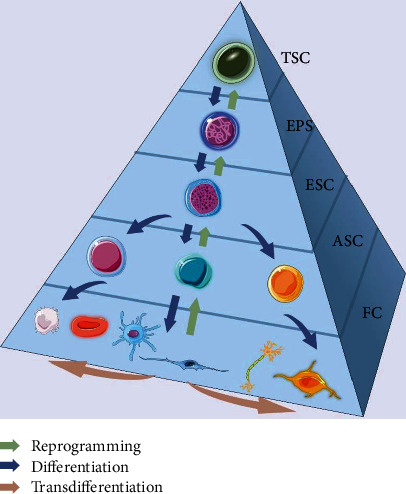
A schematic diagram for differentiation, reprogramming, and transdifferentiation. Cells come down from totipotent stem cells to functional cells in the development process (differentiation) while differentiated cells are able to be reversed back to pluripotent state (reprogramming) by transcription factors or chemical cocktails. Using similar approaches, one type of functional cells can be directly converted to other functional cells (transdifferentiation). TSC: totipotent stem cell; EPS: extended pluripotent stem cell; ESC: embryonic stem cell; ASC: adult stem cell; FC: functional cell.

Although the medical applications of iPSCs are promising, transgenic approaches raise safety concerns because of the use of oncogenes and the potential for the integration of exogenous factors. Therefore, several new methods have been developed to resolve these issues, including nonintegrating vectors, nonviral gene delivery methods, miRNAs, cell membrane permeable proteins, and small molecule compounds [7–11]. Compared to other approaches, chemical compounds similar to those employed to treat human diseases for decades have several unique advantages. For example, their structural versatility permits modulation of induction time and concentration [12]. In this review, omitting differentiation, we will focus on pure small molecule inductions for reprogramming or transdifferentiation (Figure 1). The dramatic progress in small molecule induction of cell fate decisions will undoubtedly accelerate the pace of biomedical studies and clinical translation.

## 2. Reprogramming *In Vitro*

### 2.1. Chemical-Induced Pluripotent Stem Cells (CiPSCs)

It was demonstrated that small molecules could replace transcription factors for reprogramming of iPSCs. Melton's group firstly revealed that Valproic acid (VPA) was able to promote OS-induced reprogramming of human fibroblasts [13]. Eggan's team discovered that RepSox (also named as E616452), which is an ALK5 inhibitor, could act as a substitute for Sox2 and promote reprogramming via activation of Nanog [14]. Ding's lab found that CHIR99021 and Tranylcypromine (also named Parnate) completed OK reprogramming of human somatic cells [15], and AMI-5 and A83-01 empowered Oct4-induced reprogramming of mouse fibroblasts [16]. Deng's team also found that a chemical cocktail (VPA, CHIR99021, RepSox, and Tranylcypromine) was able to reprogram mouse fibroblasts to iPSCs with Oct4 alone [17]. In 2013, Deng's team reported that mouse fibroblasts could be induced to iPSCs via a combination of seven small molecules (VPA, CHIR99021, RepSox, Tranylcypromine, Forskolin, DZNep, and TTNPB) [18]; however, this induction method has been challenged by other labs [19]. Consequently, Deng's team presented a new induction approach to resolve these problems [20]. They identified two new small molecules (AM580 and EPZ004777) to induce mouse fibroblasts into a stage named “XEN-like cell transition,” while three small molecules (5-aza-dC, EPZ004777, and SGC0946) were sufficient to convert these transitional cells to CiPSCs. Compared with the original protocol, the induction efficiency for CiPSCs was raised by 1000-fold via fine-tuning of the factors during these two stages. At the same time, Xie's team discovered that a chemical cocktail including bromodeoxyuridine (BrdU), CHIR99021, RepSox, and Forskolin was able to induce mouse fibroblasts into CiPSCs [19].

In 2016, Deng's team also reported that CiPSCs were reprogrammed from neural stem cells and intestinal epithelial cells [21]. A similar chemical cocktail (VPA, CHIR99021, RepSox, Parnate, Forskolin, AM580, and DZNep) was applied to the reprogramming of MEFs and intestinal epithelial cells. Two extra small molecules Ch55 and EPZ004777 were used in the reprogramming of neural stem cells. In 2018, Pei's team found that three types of mouse cell lineages could be induced to CiPSCs through an epithelial colony stage [22]. A chemical combination containing Vitamin C (VC), bFGF, CHIR99021, BrdU, RepSox, FSK, VPA, AM580, EPZ5676, DZNep, SGC0946, and BMP4 was applied for the induction of epithelial colonies, and then, 2iL (CHIR99021, PD0325901, and LIF) were used to induce full pluripotency in the second stage. In contrast to Deng's and Xin's methods, the induction efficiency and time were dramatically improved in Pei's protocol.

**Figure 2:**
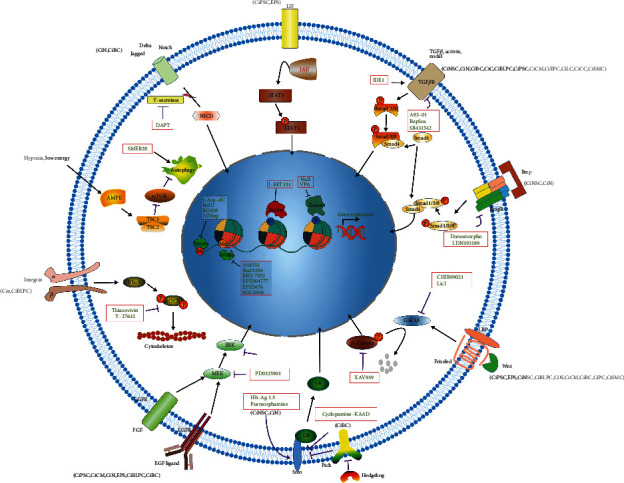
Mechanisms of small molecule induction. Small molecules targeting signaling pathways control target genes and impact cell fate decision. Small molecules also regulate epigenetic modulators modifying chromatin structure and change the epigenome and cell fate. Some other chemical compounds, such as chemicals regulating signaling activity in metabolism or cytoskeleton dynamics, also affect cell fate decision and are shown in Table 1. CiA: chemical-induced adipocyte; CiBC: chemical-induced beta cell; CiBLPC: chemical-induced bipotent liver progenitor cell; CiCC: chemical-induced cartilaginous cell; CiCM: chemical-induced cardiomyocyte; CiEC: chemical-induced epithelial colony; CiEPC: chemical-induced endodermal progenitor cell; CiLC: chemical-induced Leydig cell; CiN: chemical-induced neuron; CiNPC: chemical-induced neuroprogenitor cell; CiNSC: chemical-induced neural stem cell; CiPSC: chemical-induced pluripotent stem cell; CiSMC: chemical-induced skeletal muscle cell; EPS: extended pluripotent stem cell.

Although mouse CiPSCs have advanced in the last several years, generation of human CiPSCs have remained elusive. Based on different pluripotent signaling pathways in mice and humans [23, 24], a large-scale screening of small molecules may be necessary. Currently, the small molecules involved in induction are classified into three categories, including epigenetics, signaling pathways, and metabolism (Table 1 and Figure 2). As for different starting cells and targeted cells, some clues could be obtained to select small molecules for reprogramming or transdifferentiation from this review.

### 2.2. Extended Pluripotent Stem Cells (EPSs)

In 2017, Deng's team found that ESCs or iPSCs could be reprogrammed into extended pluripotent stem cells (EPSs) that could differentiate into four lineages including trophectoderm, ectoderm, endoderm, and mesoderm via a chemical cocktail consist of LIF, CHIR99021, (S)-(+)-Dimethindene maleate, and Minocycline hydrochloride [25]. After half a year, Liu's team also obtained EPSs using a different small molecule combination containing hLIF, CHIR99021, PD0325901, JNK inhibitor VIII, SB203580, A-419259, and XAV939 [26]. As for a means to create new animal models, EPS cell lines could be applied to explore fundamental questions such as the development of the placenta, yolk sac, and embryo proper.

### 2.3. Chemical-Induced Neural Stem Cells (CiNSCs)

In 2012, we first found a pure small molecule combination (VPA, RG108, VC, BIX01294, A83-01, CHIR99021, and PD032591) was able to induce mouse embryonic and adult tail-tip fibroblasts into neural stem cells [27, 28]. CiNSCs are similar to neural stem cells in morphology, gene expression patterns, self-renewal capacity, excitability, and multipotency. Moreover, they can be differentiated into three types of nerve cell lineages *in vitro* and *in vivo*. Based on the same small molecule combination, Pei's team also recently obtained CiNSCs from mouse fibroblasts in hypoxia (5% O_2_) [29]. In 2016, Ding's team revealed a new approach to induce mouse fibroblasts into induced neural stem cells (CiNSCs) using a combination of nine small molecules (M9), including LDN193189, A83-01, CHIR99021, bFGF, Hh-Ag 1.5, retinoic acid, RG108, Parnate, and SMER28 [30]. Specific transcription factors Elk1 and Gli2 were upregulated by M9 treatment, which, in turn, upregulated expression of the endogenous master neural gene Sox2 to complete induction.

### 2.4. Chemical-Induced Bipotent Liver Progenitor Cells (CiBLPCs)

In 2017, Ochiya's lab converted mature rat and mouse hepatocytes into bipotent liver progenitor cells with three small molecules (Y-27632, A83-01, and CHIR99021) *in vitro* [31]. Although the reprogramming methods were efficient in rats and mice, they failed in humans. In 2018, Hui's team induced human hepatocytes into bipotent liver progenitor cells using four small molecules A83-01, Y-27632, CHIR99021, and Wnt3a [32]. Later, two research teams obtained human bipotent liver progenitor cells from hepatocytes with different methods such as Y-27632, CHIR99021, A83-01, S1P, and LPA [33] and A83-01, CHIR99021, EGF, and HGF [34], respectively.

### 2.5. Chemical-Induced Endodermal Progenitor Cells (CiEPCs)

In 2016, Pei's team revealed that human gastric epithelial cells could be reprogrammed to endodermal progenitors with a small molecule cocktail (Bay-K-8644, Bix01294, RG108, and SB431542) used to treat tissue-specific mesenchymal feeders [35]. The resulting chemical-induced endodermal progenitors were able to be amplified in culture and differentiated to hepatocytes, pancreatic endocrine cells, and intestinal epithelial cells without generation of teratomas *in vivo*.

Although the mechanism of small molecule induction remains elusive, some clues can be obtained from the current literature. Taken together, to complete reprogramming, the epigenetic barrier has to be overcome and the starting cell identity should be gradually removed, while the target cell identity should be built up. In pure small molecule reprogramming, epigenetic modulators such as DNA methylation writer inhibitors (5-aza-dC, BrdU, DZNep, and RG108), histone methylation writer inhibitors (Bix01294, EPZ004777, EPZ5676, and SGC0946), and histone acetylation eraser inhibitors (VPA) were involved in this process (Table 1 and Figure 2). If fibroblasts are the starting cells, the TGF-*β* signaling pathway needed to be shut down by chemicals (SB431542, A83-01, and RepSox), which indicates this pathway is essential to keep the identity of fibroblasts. To create target cell identity, the Wnt signaling pathway needed be activated to reverse the induced cells back to an earlier developmental stage during reprogramming with an activator (CHIR99021). Due to cell death caused by oxidative stress and an epigenetically unstable state during the reprogramming process, metabolic regulators ((S)-(+)-Dimethindene maleate, Vitamin C, Parnate, Ch55, SMER28, AM580, and TTNPB) have been applied to enhance cell survival during the conversion.

## 3. Transdifferentiation *In Vitro*

Pluripotent stem cells (ESCs and iPSCs) should be converted into functional target cells before injection for cell therapy because they could generate teratomas *in vivo* [36]. The technology of transdifferentiation (i.e., the transition from one functional cell type to another without a requirement of a pluripotent state) represents a shortcut to achieve sufficiently functional cells for cell therapy [37]. At present, several types of functional cells including neurons, photoreceptor cells, cardiomyocytes, beta cells, adipocytes, skeletal muscle cells, cartilaginous cells, and Leydig cells have been successfully obtained using small molecule-mediated transdifferentiation methods *in vitro*.

### 3.1. Chemical-Induced Neurons (CiNs)

As life expectancy is increasing, the number of people suffering from neurodegenerative disorders such as Alzheimer's and Parkinson's disease is on the rise [38]. Thus, it is urgent to obtain adequate quantities of patient-tailored neural cells for cell therapy and drug screening. Nowadays, scientists have made great progress in small molecule-based direct induction for neurons. In 2015, Deng's team used a combination of four small molecule compounds (Forskolin, ISX9, CHIR99021, and I-BET151) to transdifferentiate mouse fibroblasts into neurons [39]. The authors suggested that I-BET151 (a BET family bromodomain inhibitor) disrupted the fibroblast-specific program, while ISX9 (a neurogenesis inducer) activated neuronal-specific genes. At the same time, Pei's work revealed that human fibroblasts were able to transdifferentiate into neurons by a different chemical cocktail (VPA, CHIR99021, RepSox, Forskolin, SP600125, Gö6983, and Y-27632) [40]. It was also reported that human lung fibroblasts could be converted into neurons using a similar small molecule combination, including VPA, CHIR99021, DMH1, RepSox, Forskolin, Y-27632, and SP600125 [41].

In 2019, Dai's research group found a rapid and efficient method to convert human fibroblasts into neurons with twelve small molecules (CHIR99021, LDN193189, Dorsomorphin, ISX9, RG108, PD0325901, Purmorphamine, DAPT, Forskolin, ISX9, Y-27632, and P7C3) [42].

In 2015, Chen's team identified a combination of nine small molecules (LDN193189, SB431542, TTNPB, Thiazovivin, CHIR99021, VPA, DAPT, Smoothened agonist, and Purmorphamine) for reprogramming human astrocytes into neurons [43]. These induced neurons could survive for more than 5 months in culture and generated functional synaptic networks *in vitro*, and they were able to survive for over 1 month in mouse brains and merge with local circuits. Later, they also implied that six signaling pathways including SHH, Notch, Wnt, BMP, TGF-*β*, and JA/STAT played a pivotal role during the transdifferentiation [44]. Similar work was reported by Pei's lab with a different small molecule combination (VPA, Chir99021, RepSox, Forskolin, I-Bet151, and ISX-9) two years later [45].

Furthermore, subtype neurons also have been obtained. In 2018, human and mouse motor neurons were created by a chemical combination containing Kenpaullone, Forskolin, Y-27632, Purmorphamine, and retinoic acid [46]. One year later, Li's team reported that a chemical cocktail (CHIR99021, A83-01, Y-27632, VPA, TTNPB, Forskolin, and NaB) induced human urine-derived cells into neurons, while the majority of induced cells were glutamatergic neurons [47].

### 3.2. Chemical-Induced Photoreceptor Cells (CiPCs)

Vision loss resulting from retinal neuron damage causes retinopathies, including age-related macular degeneration, diabetic retinopathy, and retinitis pigmentosa [48, 49]. As a favorable method, stem cell therapy could substitute for the loss of retinal neurons [50, 51]. Recently, Chavala's team reported five small molecules (VPA, CHIR99021, RepSox, Forskolin, and IWR1) were able to transdifferentiate fibroblasts into photoreceptor-like cells [52]. The authors also confirmed that CiPCs could mend pupil reflex and vision when transplanted into the subretinal space of mice with retinal degeneration. Additionally, they implied that the AXIN2–NF-*κ*B–ASCL1 pathway enhanced retinal lineage commitment and mitochondria were the signaling hub during transdifferentiation.

### 3.3. Chemical-Induced Cardiomyocytes (CiCMs)

It is widely known that the regeneration of the adult mammalian heart after injury is limited [53]. Therefore, heart failure resulting from cardiomyocyte loss is a major cause of mortality around the world [54]. As the most common cell type in the heart, cardiac fibroblasts are considered promising for cardiac reprogramming.

Small molecules are also able to replace transcription factors and provide an alternative means of cardiac reprogramming. It was reported that TGF-*β* inhibitors (SB431542 or A83-01) could improve the efficiency of cardiomyocyte induction [55–57]. The small molecule Y-27632 also enhanced cardiac reprogramming [58]. Furthermore, Ding's group reported that a small molecule combination (CHIR99021, SB431542, Parnate, and Forskolin) was sufficient to complete the conversion of cardiomyocytes from mouse fibroblasts with Oct4 alone [59]. It was also reported that small molecules (NaB, RA, and ICG-001) were able to improve rat and human cardiac cell generation induced by transcription factors (Gata4, Mef2C, and Tbx5) [60]. In 2015, Xie's team transdifferentiated mouse fibroblasts into cardiomyocytes by passing a cardiac progenitor stage with six small molecules (CHIR99021, RepSox, Forskolin, VPA, Parnate, and TTNPB), while the induced cardiomyocytes were cultured in cardiomyocyte maintenance medium containing CHIR99021, PD0325901, LIF, and insulin [61]. One year later, Ding's lab reported that human functional cardiomyocytes were induced by a combination of nine small molecules (CHIR99021, A83-01, BIX01294, AS8351, SC1, Y-27632, OAC2, SU16F, and JNJ10198409) [62]. Furthermore, the induced human fibroblasts were able to be efficiently converted into cardiomyocyte-like cells in infarcted mouse hearts.

### 3.4. Chemical-Induced Beta Cells (CiBCs)

Diabetes mellitus, which results from pancreatic *β* cell damage, is an international health epidemic and influences more than 300 million people in the world [63]. Therefore, producing plenty of functional pancreatic *β* cells for studying diabetes and treating patients is an urgent task. In 2015, we successfully induced human urine cells to insulin-secreting beta cells by passing through three stages with pure small molecules [64]. Firstly, urine cells were induced into an endodermal lineage using a chemical cocktail (IDE 1, LiCl, and VC) for 6 days. The induced cells were then differentiated into pancreatic precursors in two steps. The first step induction medium contained cyclopamine-KAAD, Indolactam V, RA, VC, A83-01, and BRD 7552 for 1 day, while the secondary step induction used chemicals, including cyclopamine-KAAD, Indolactam V, VC, A83-01, and BRD 7552, for 6 days. Insulin-secreting beta cells were obtained in the tertiary induction medium (SB203580, VC, and DAPT) for 9 days. Furthermore, the induced beta cells could reduce glucose levels and enhance survival rates in diabetic mice.

### 3.5. Chemical-Induced Adipocytes (CiAs)

As a promising therapy for obesity and metabolic diseases, brown adipose tissue (BAT) has been intensively studied [65, 66]. The energy balance in the body is balanced with white adipose tissue collecting energy, while BAT expends energy and produces heat [67]. In 2017, Ding's research group converted mouse myoblasts into brown adipocyte-like cells with a retinoid X receptor (RXR) agonist, bexarotene. They implied that Rxr*α*/*γ* activation is required for the induction of BAT [68].

### 3.6. Chemical-Induced Skeletal Muscle Cells (CiSMCs)

Muscle-related maladies including muscle wasting and muscular dystrophy have yet-to-be adequately treated using traditional medicine. The cell therapy technique brings a promising approach to resolve this issue. Recently, it was reported that mouse fibroblasts could be converted to skeletal muscle cells by a combination of six small molecules (VPA, Chir99021, RepSox, Forskolin, Parnate, and TTNPB) [69]. The authors implied that three signaling pathways Wnt, TGF-*β*, and cAMP were crucial for the transdifferentiation.

### 3.7. Chemical-Induced Cartilaginous Cells (CiCCs)

Cartilage defects cause joint pain and diminish quality of life. Recently, autologous chondrocyte therapy was proposed as a means of cartilage healing [70]. Ouyang's team revealed that mouse embryonic fibroblasts could be converted to functional cartilaginous cells by a chemical cocktail (VPA, CHIR98014, RepSox, TTNPB, and Celecoxib) [71]. These CiCCs could enhance defective healing and restore 63.4% of mechanical function damage *in vivo*.

### 3.8. Chemical-Induced Leydig Cells (CiLCs)

Affecting about 30% of men aged 40–79 years, late-onset hypogonadism (LOH) with a serum testosterone deficiency could result in sexual dysfunction, central adiposity, mood disturbance, osteoporosis, amyotrophy, and other abnormalities [72–74]. Leydig cells produce testosterone, so Leydig cell transplantation could be an ideal tool to heal LOH. Recently, Huang's team reported that functional mouse Leydig cells could be transdifferentiated from fibroblasts using a small molecule combination (Forskolin, 20a-hydroxycholesterol, luteinizing hormone, and SB431542) [75]. Moreover, these CiLCs could survive in the testes and produce testosterone in a circadian rhythm.

As for the mechanism of small molecule transdifferentiation, collectively, in contrast to reprogramming, transdifferentiation is an easier process because it does not need more energy to pull the starting cells to a less differentiated level for cell conversion. Compared to reprogramming, epigenetic modulators, the histone methylation writer inhibitor was replaced with the histone acylation reader inhibiter (I-Bet151) in transdifferentiation, which implies less epigenetic barrier is required to be overcome during transdifferentiation. Furthermore, more metabolic modulators are involved in the confirmation of the new cell identity, such as OAC2 for cardiomyocytes, ISX9 for neurons, and bexarotene for brown adipose tissue.

## 4. Transdifferentiation *In Vivo*

Although functional cells could be obtained by differentiation from pluripotent stem cells or transdifferentiation from somatic cells, induction efficiency, ultimate maturation of cells, and survival rates after cell transplantation are still the three biggest obstacles to cell therapy [76]. Due to safety and technical difficulties of cell transplantation therapy, *in vivo* reprogramming may become the next generation of regenerative medicine with therapeutic potential [77].

### 4.1. Neurons

In 2018, Deng's team released their data about *in vivo* transdifferentiation of neurons from mouse astrocytes with a cocktail combination consist of dbcAMP, Forskolin, ISX9, CHIR99021, I-BET151, and Y-27632 [78]. The combination of chemicals was injected into mouse brains at a stable rate for two weeks with an osmotic minipump. The induced cells not only formed endogenous neurons with similar neuron-specific marker expression and electrophysiological properties but also merged with local circuits *in vivo*.

### 4.2. Cardiomyocytes

In 2018, Xie's team reported that a small molecule combination of CRFVPTM (CHIR99021, RepSox, Forskolin, VPA, Parnate, TTNPB, and Rolipram) mediated transdifferentiation of cardiac fibroblasts into cardiomyocytes in normal adult mice with a low efficiency of 1% [79]. CRFTM were administrated orally and VP were intraperitoneally injected once for 6 weeks. The transdifferentiation only happened in the heart, which suggests the local niche also plays a critical role in small molecule-mediated cardiac induction. Furthermore, the induced cardiomyocytes dramatically repressed the scar formation and promoted cardiac function in mice with a myocardial infarction.

To explore the mechanism of small molecule transdifferentiation *in vivo* and compare transdifferentiation *in vitro* and *in vivo*, additional small molecules were applied to activate the cAMP signaling pathway (dbcAMP for neurons and Rolipram for cardiomyocytes), which suggested targets downstream of the PKA signaling pathway are important to overcome the disturbance from *in vivo* environment during transdifferentiation.

In summary, although the mechanism of full small molecule induction is unknown, some implications can be observed. By examining signaling pathways, it is apparent that certain pathways are preferred for transdifferentiation (Figure 2), such as inhibiting BMP for ectodermal induction, activation of LIF-STAT3 for creating pluripotent stem cells, and inhibition of Notch, SHH, and Rho for the induction of ectodermal or endodermal lineages. On the other hand, some signaling pathways are preferred for induction (e.g., activation of Wnt and inhibition of TGF-*β* and MAPK/ERK). As for the induction process, it seems that there is an intermediate state by which various target cells could be achieved in certain culture conditions.

## 5. Perspective

Despite the exciting progress that has been achieved in the field of pure small molecule-induced cells, there are still some key problems such as apoptosis due to oxidative stress, death from an epigenetically unstable state, genomic integrity, genotoxicity, scaling production for large animals' safety and efficacy trials, and producing a safe delivery system as well as induction methods [77]. Moreover, the majority of pure small molecule cocktails for human cells still remain to be determined.

**Figure 3:**
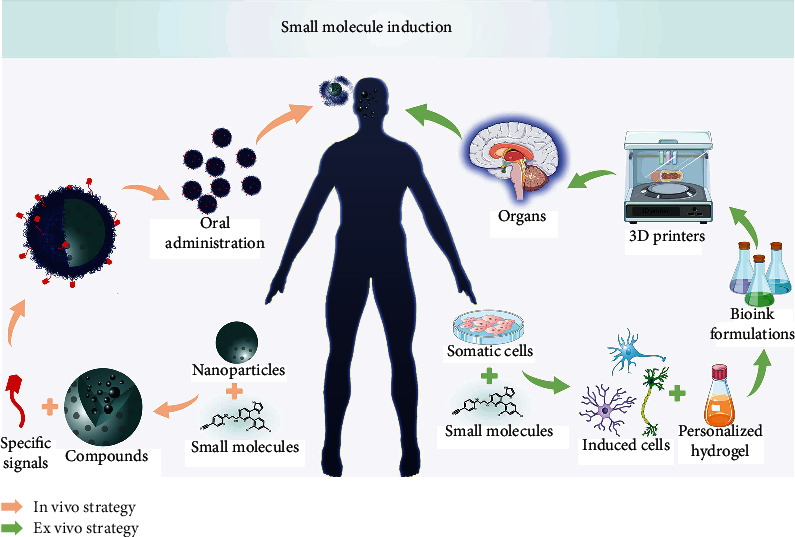
Future trends for small molecule-mediated personalized cell therapy. On the one side, somatic cells will be transdifferentiated to functional cells *in vitro* and then organized to organs by a 3D printer, and the personalized organs will be transplanted into patients finally. On the other side, nanoparticles carrying small molecule cocktails target specific cells for in situ induction *in vivo*.

Without cell transplantation, direct *in vivo* reprogramming for local *in situ* conversion of cells is emerging as a new way to produce cells for regenerative medicine. Although *in situ* chemical induction will be a focus for the next decade, how these small molecules could be precisely delivered to the desired tissues or organs to produce fully integrated functional cells is a primary challenge. Biomaterials that can deliver small molecules to targeted organs, for example, nanoparticles containing specific signals for recognizing specific cell types, can assist *in vivo* reprogramming studies and future clinical applications (Figure 3). On the other hand, small molecule-induced cells could be constructed for organs such as the heart, liver, or brains using 3D printers *in vitro* (Figure 3). In addition, recent scientific tools such as single-cell sequencing [80] and CRISPR-based genome-wide screening [81] will help exploring new chemical cocktails and illustrate the induction mechanisms.

## Figures and Tables

**Table 1:** Small molecules involved in pure small molecule-induced reprogramming or transdifferentiation.

Name of the compounds	Main mechanism of action	Application in reprogramming or transdifferentiation	References
*Signaling pathways*			
*TGF-β signaling pathways*			
A83-01	TGF-beta RI (ALK4/5/7) inhibitor	CiNSCs, CiNs, CiBCs, CiPSCs, CiBLPCs, CiCMs, CiEPCs	[16, 27, 28, 30–34, 46, 55–57, 62, 64]
RepSox (E-616452)	TGF-beta RI (ALK5) inhibitor	CiPSCs, CiNs, CiCMs, CiPCs, CiSMCs, CiCCs	[14, 17–19, 21, 22, 40, 41, 45, 52, 61, 69, 71, 79]
SB431542	Inhibitor of TGF-*β*RI, ALK4, and ALK7	CiEPCs, CiNs, CiCMs, CiLCs	[35, 43, 55–57, 59, 75]
IDE 1	Activator of TGF-*β* signaling pathway	CiBCs	[64]
DMH1	Inhibitor of ALK2	CiNs	[41]
*BMP signaling pathways*			
Dorsomorphin	BMP receptor inhibitor	CiNs	[42]
LDN193189	BMP type I receptor (ALK2/3) inhibitor	CiNSCs, CiNs	[30, 42, 43]
*Wnt signaling pathway*			
CHIR99021	GSK3 inhibitor	CiPSCs, EPSs, CiNSCs, CiBLPCs, CiNs, CiCMs, CiPCs, CiSMCs	[15, 17–19, 21, 22, 25–28, 30–34, 39–43, 45, 47, 52, 59, 61, 62, 69, 78, 79]
LiCl	GSK3 inhibitor	CiBCs	[64]
XAV939	Wnt/beta-catenin inhibitor	EPSs	[26]
IWR1	Wnt/beta-catenin inhibitor	CiPCs	[52]
CHIR-98014	GSK3 inhibitor		
TWS119	GSK3 inhibitor		
Tideglusib	GSK3 inhibitor		
BIO	GSK3 inhibitor		
AZD2858	GSK3 inhibitor		
TDZD-8	GSK3 inhibitor		
Indirubin	GSK3 inhibitor		
PNU-74654	Wnt/beta-catenin inhibitor		
IWP-2	Wnt/beta-catenin inhibitor		
*MAPK/ERK signaling pathway*			
PD0325901	Inhibitor of MEK1/2	CiPSCs, CiCMs, CiNs, EPSs	[22, 26, 42, 61]
SC1	ERK1 and RasGAP inhibitor	CiCMs	[62]
*Rho signaling pathway*			
Thiazovivin	ROCK inhibitor	CiNs	[43]
Y-27632	ROCK inhibitor	CiNs, CiBLPCs, CiCMs	[31–33, 40, 42, 46, 47, 58, 62, 78]
*Notch signaling pathway*			
DAPT	Gamma-secretase inhibitor	CiNs, CiBCs	[42, 43, 64]
*SHH signaling pathway*			
Cyclopamine-KAAD	Hedgehog/smoothened inhibitor	CiBCs	[64]
Hh-Ag 1.5	Smoothened agonist	CiNSCs	[30]
Purmorphamine	Smoothened agonist	CiNs	[42, 43, 46]
*Other signaling pathways*			
A-419259	An inhibitor of Src family kinases (SFK)	EPS	[26]
dbcAMP	Activates cAMP-dependent protein kinases		[78]
Forskolin	Adenylyl cyclase activator	CiPSCs, CiNs, CiPCs, CiSMCs, CiLCs	[18, 19, 22, 39–42, 45–47, 52, 59, 61, 69, 75, 78, 79]
Gö6983	Inhibitor of protein kinase C (PKC)	CiNs	[40]
Indolactam V	Activator of protein kinase C (PKC)	CiBCs	[64]
JNJ10198409	PDGFR-a and PDGFR-b inhibitor, PDGFR tyrosine kinase inhibitor IV	CiCMs	[62]
SB203580	P38 MAPK inhibitor	EPSs, CiBCs	[26, 64]
SP600125	JNK inhibitor	CiNs	[40, 41]
SU16F	PDGFR-b inhibitor	CiCMs	[62]
Celecoxib	COX inhibitor	CiCCs	[71]
*Epigenetic modifications*			
*DNA methylation inhibitor*			
5-Aza-dC	DNMT inhibitor	CiPSCs	[20]
BrdU (bromodeoxyuridine)	Analog of thymidine	CiPSCs	[19, 22]
DZNep	SAH hydrolase inhibitor	CiPSCs	[18, 21, 22]
RG108	DNA methyltransferase inhibitor	CiNSCs, CiEPCs, CiNs	[27, 28, 30, 35, 42]
AMI-5	Protein methyltransferase inhibitor	CiPSCs	[16]
PF-6405761	BET inhibitor		
*Histone deacetylation inhibitor*			
NaB	HDAC inhibitor	CiNs, CiCMs	[47, 60]
VPA	HDAC inhibitor	CiPSCs, CiNSCs, CiNs, CiCMs, CiPCs, CiSMCs, CiCCs	[13, 17, 18, 21, 22, 27, 28, 40, 41, 43, 45, 47, 52, 61, 69, 71, 79]
I-BET-762	BET inhibitor		
*Histone methylation modulator*			
AS8351	Inhibitor of histone demethylase	CiCMs	[62]
Bix01294	Histone methyltransferase inhibitor	CiCMs, CiEPCs, CiNSCs	[27, 28, 35, 62]
BRD 7552	Increases acetylation of histone H3 and trimethylation of H3K4 and H3K9	CiBCs	[64]
EPZ5676	DOT1 inhibitor	CiPSCs	[22]
EPZ004777	DOT1L inhibitor	CiPSCs	[20, 22]
SGC0946	DOT1L inhibitor	CiPSCs	[20, 22]
CPI-0610	BET inhibitor		
GS-5829	BET inhibitor		
*Histone acetylation modulator*			
I-BET151	Inhibitor of epigenetic reader	CiNs	[39, 45, 78]
INCB057643	BET inhibitor		
*Metabolic processes*			
AM580	RAR agonist	CiPSCs	[20–22]
Bexarotene	RAR agonist	CiAs	[68]
Ch55	RAR agonist	CiPSCs	[21]
Retinoic acid	RAR ligand	CiNSCs, CiNs	[30, 46]
TTNPB	RAR ligand	CiPSCs, CiNs, CiSMCs, CiCCs	[18, 43, 47, 61, 69, 71]
Bay-K-8644	Ca2+ channel activator	CiEPCs	[35]
ISX9	Neurogenesis inducer	CiNs	[39, 42, 78]
LPA	A ligand activator for EDG-2, EDG-4, and EDG-7	CiBLPCs	[33]
Minocycline hydrochloride	Bind to the bacterial 30S ribosomal subunit and inhibiting protein synthesis	EPSs	[25]
OAC2	Activator of octamer-binding transcription factor 4 (Oct4)	CiCMs	[62]
Parnate (Tranylcypromine)	Monoamine oxidase inhibitor, LSD1 inhibitor	CiPSCs, CiNSCs, CiCMs, CiSMCs	[15, 17, 18, 21, 30, 59, 61, 69, 79]
P7C3	Targets NAMPT enzyme	CiNs	[42]
Rolipram	PDE4 inhibitor		[79]
SMER28	Autophagy modulator	CiNSCs	[30]
(S)-(+)-Dimethindene maleate	Antagonist of muscarinic M2 and histamine H1 receptors	EPSs	[25]
Vitamin C	A strong antioxidant	CiPSCs, CiNSCs, CiBCs	[22, 27, 28, 64]

CiAs: chemical-induced adipocytes; CiBCs: chemical-induced beta cells; CiBLPCs: chemical-induced bipotent liver progenitor cells; CiCCs: chemical-induced cartilaginous cells; CiCMs: chemical-induced cardiomyocytes; CiECs: chemical-induced epithelial colonies; CiEPCs: chemical-induced endodermal progenitor cells; CiLCs: chemical-induced Leydig cells; CiNs: chemical-induced neurons; CiNPCs: chemical-induced neuroprogenitor cells; CiNSCs: chemical-induced neural stem cells; CiPCs: chemical-induced photoreceptor cells; CiPSCs: chemical-induced pluripotent stem cells; CiSMCs: chemical-induced skeletal muscle cells; EPSs: extended pluripotent stem cells.
